# Postural analysis of children with muscle retraction after six-month exercise or heel cup interventions in a randomized trial

**DOI:** 10.1038/s41598-025-98527-6

**Published:** 2025-04-19

**Authors:** Vicenta Martínez-Córcoles, Jonatan García-Campos, Juan Vicente-Mampel, Esther Chicharro-Luna, Eva María Martínez Jiménez, Javier Ferrer-Torregrosa

**Affiliations:** 1 Department of Behavioral Sciences and Health, Miguel Hernandez University, Alicante, Spain; 2 Institute for Health and Biomedical Research (ISABIAL), Alicante, Spain; 3 Department of Physiotherapy, School of Medicine and Health Sciences, Valencia Catholic University “San Vicente Mártir”, Torrent, Valencia Spain; 4 Nursing, Physiotheray and Podiatrist Department, Complutense University of Madrid, Madrid, Spain; 5 Department of Podiatry, School of Medicine and Health Sciences, Valencia Catholic University “San Vicente Mártir”, Valencia, Spain

**Keywords:** Exercise, Postural control, Children, Heel cup, Shortening, Triceps Surae, Diseases, Health care

## Abstract

Balance and proprioception are crucial in children, especially in sports. The ankle joint plays a key role in postural control, and triceps surae retraction is common in active children. This study aimed to analyze the effects of stretching exercises versus heel cups on postural control in children aged 8 to 12 with triceps surae retraction. A six-month randomized controlled trial was conducted with 150 children assigned to either stretching exercises (*n* = 76) or heel cups (*n* = 74). The study was registered at ClinicalTrials.gov under the identification number NCT05902949 (first posted on 06/15/23). The area of oscillation was measured using the Gyko system under four conditions: eyes open/closed and firm ground/foam. A linear mixed regression model was applied to analyze the effects of the treatments, adjusting for sex, age, and physical activity. Stretching exercises were associated with significant reductions around oscillation across all conditions (*p* < 0.001 at six months). In contrast, heel cups were linked to significant increases around oscillation in all conditions (*p* < 0.001 at six months), indicating a negative effect on balance. Additionally, higher levels of physical activity and female sex were associated with better postural control (*p* < 0.001). In conclusion, stretching exercises significantly improved postural control in children with triceps surae retraction, whereas heel cups worsened this essential motor-performance ability. Further research is needed, but these findings support the incorporation of stretching exercises and regular physical activity in the management of these patients.

**Clinical trial registration number**: NCT05902949.

**Supplementary Information:**

The online version contains supplementary material available at 10.1038/s41598-025-98527-6.

## Introduction

Body movements and their execution are crucial in balance studies, especially concerning lower limb injuries in sports^1,2^. During childhood, the neuromuscular system develops continuously; thus, enhancing proprioception and balance early can significantly prevent injuries and aid motor development. Exercise programs aimed at improving proprioception and balance can strengthen stabilizing muscles and enhance coordination. Additionally, postural control and stability analyses are used in medical and clinical fields for various rehabilitative treatments and injury prevention^1–3^.

Balance coordination involves the central nervous system (CNS), and the visual, vestibular, and proprioceptive systems, which are responsible for muscle activation^4^. Notably, muscle activation in postural control is critical at the ankle joint^5,6^. Ankle proprioception, along with the gastrocnemius and soleus muscles, is vital for maintaining postural control in sports^6–8^. García-Soidán et al. (2020) found that children engaged in sports enhance their postural stability and sensory maturation^9^. Physical exercise in children influences the development of lower limb muscles, particularly at the ankle and foot^9^. Calcaneal apophysitis, a leading cause of heel pain in children aged 8–12 years, has an incidence of 3.7 per 1000 children, with frequent occurrences in sports consultations. This condition is marked by inflammation of the calcaneus.

Children involved in sports like soccer, basketball, or track and field face a higher risk of developing calcaneal apophysitis due to repetitive stress on the heel. This pain typically worsens during physical activity, potentially limiting sports participation. Children with muscle retraction and calcaneal apophysitis often need to modify their activities, rest, perform proper stretching, and sometimes use orthotic footwear (heel cups) to alleviate heel pressure. Properly addressing this condition is crucial to prevent long-term complications and enable safe sports participation. This study aims to analyze postural control in school-aged children (8 to 12 years old) with triceps surae muscle retraction using stabilometric tests to compare the effectiveness of stretching treatments versus heel inserts over 3 to 6 months.

## Materials and methods

### Study design

A single-blind 28-week randomized controlled trial (RCT) was performed between 28-06-2023 and 28-12-2023 with two parallel experimental groups. This study was approved by the Research Ethics Committee of the Universidad Católica de Valencia (UCV/2017–2018/113) in accordance with the ethical guidelines of the Helsinki Declaration, 2018. In addition, the study was registered at Clinicaltrial.gov and assigned the identification number NCT05902949 (first posted date: 15/06/23). The informed consent was obtained from all participants before the start of the study.

### Blinding and allocation

This assignment was performed using a computer-generated block randomization process. All patients were treated by a physiotherapist and two podiatrists with extensive experience (> 10 years) in the treatment employed. The second physiotherapist was blinded to the group being assessed. As it is impossible to blind the participants and the treating physiotherapist to the application of the heel cup and stretching exercise, a single-blind design was chosen.

### Study populations

**Fig. 1:**
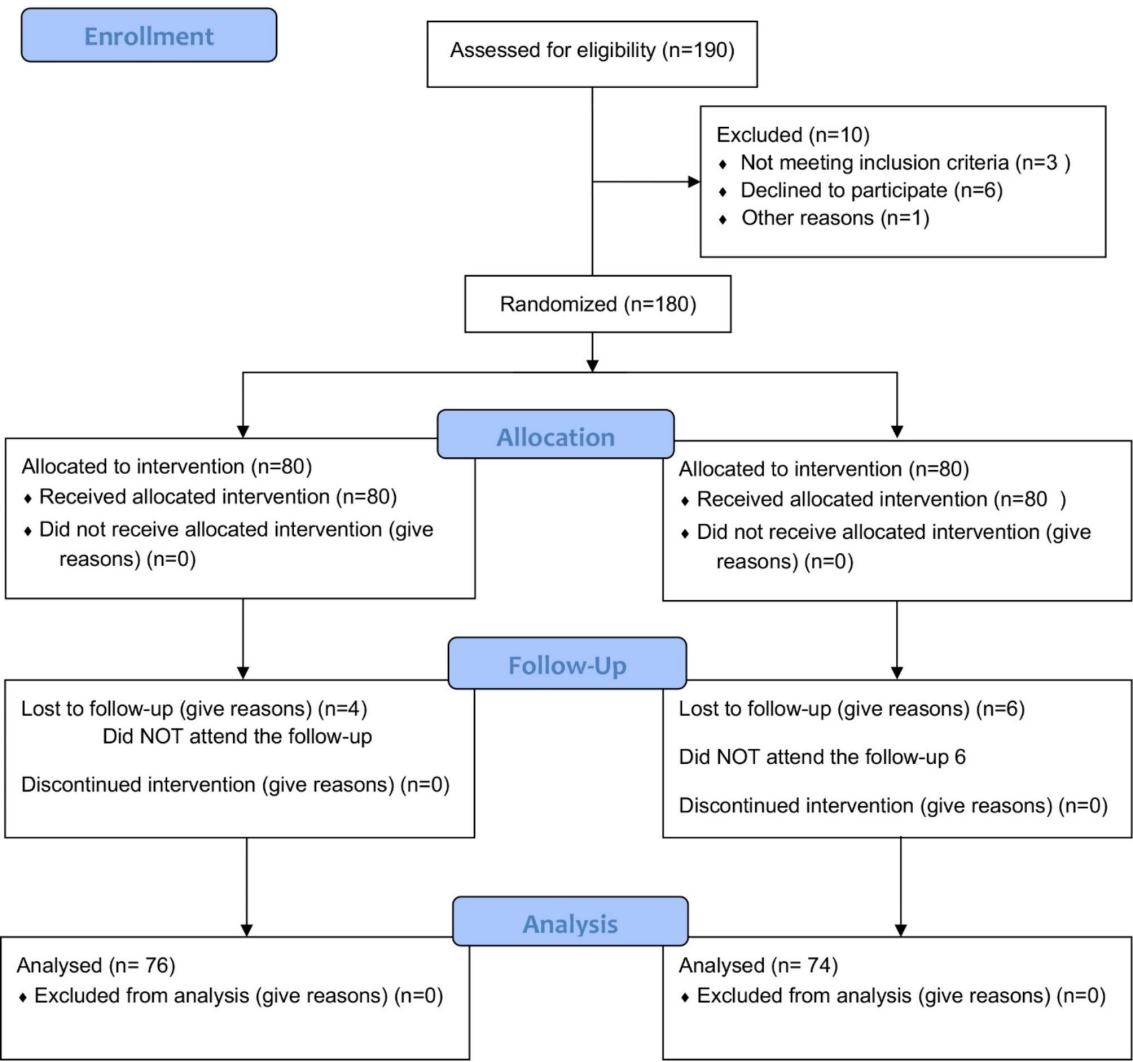
The design and progression of participants through the trial were conducted.

A total of 150 children participated in this study. The study is presented in Fig. 1 and the descriptive values are shown in Table 1 The study is presented in Fig. 1 and the descriptive values are shown in Table 1; Fig. 1. All participants were recruited from primary schools in the provinces of Albacete and Alicante (Spain). Inclusion criteria were: (i) Children aged 8–12 years old; (ii) regular sporting activity; and (iii) decreased range of flexion < 10 cm in the lunge test [10]. The exclusion criteria were as follows: (i) neurological, vestibular, muscular, psychological, or visual disease; and (ii) traumatic pathology 12 months prior to the investigation measurement (sprains, heel pain, etc.). (iii) Diseases of balance or motor control, (iv) surgeries in the last 12 months, (v) taking medications that may affect the neuromuscular system, and (vi) participation in sports in the last 48 h.

**Table 1:** Demographic data.

	All *n* = 150	Exercises *n* = 76	Heel pad *n* = 74	Pvalue
Age (years)	9.91 ± 1.23	9.91 ± 1.22	9.91 ± 1.25	0.96
Sex				
Male (n,%)	83 (55.33%)	38 (45.78%)	45 (54.22%)	0.34
Female (n,%)	67 (44.67%)	36 (53.73%)	31 (46.27%)	
Height (m)	133.29 ± 13.70	133.04 ± 17.25	133.55 ± 8.78	0.82
Weight (kg)	37.83 ± 9.58	38.12 ± 9.17	37.52 ± 10.03	0.70
Body mass index (kg/m^2^)	20.62 ± 3.04	20.61 ± 2.95	20.63 ± 3.15	0.98
Thin (n.%)	7 (4.67%)	3 (3.95%)	4 (5.4%)	0.69
Normal (n.%)	95 (63.33%)	48 (63.16%)	47 (63.5%)	
Obese (n.%)	19 (12.67%)	8 (10.53%)	11 (14.9%)	
Overweight (n.%)	29 (19.33%)	17 (22.37%)	12 (16.2%)	
Right footprint				
Highly pronated (n.%)	1 (0.67%)	1 (1.32%)	–	o.25
Highly supinated (n.%)	35 (23.33%)	22 (28.95%)	13 (17.6%)	
Normal (n.%)	52 (34.67%)	24 (31.58%)	28 (37.8%)	
Prone (n.%)	50 (33.33%)	22 (28.95%)	28 (37.8%)	
Supinated (n.%)	12 (12%)	7 (9.21%)	5 (6.8%)	
Left footprint				
Highly pronated (n.%)	1 (0.67%)	1 (1.32%)	–	0.33
Highly supinated (n.%)	35 (23.33%)	22 (28.95%)	13 (17.6%)	
Normal (n.%)	55 (36.67%)	27 (35.53%)	28 (37.8%)	
Pronated (n.%)	47 (31.33%)	19 (29.00%)	28 (37.8%)	
Supinated (n.%)	12 (8.00%)	7(9.21%)	5 (6.8%)	

Data on age, height, weight and body mass index (BMI) are presented as mean ± standard deviation (n ± sd). Categorical variables, such as sex, BMI classification and plantar footprint types, are expressed as absolute frequencies and percentages (n, %). Differences between groups were analyzed by statistical tests, and p values indicate the significance of such comparisons.

### Primary outcomes

#### Stabilometry

The standard protocol proposed by the International Society for the Advancement of Kinanthropometry (ISAK, 2001) was used to assess balance. The participants stood barefoot with their feet 4 cm apart and externally rotated 30°, keeping the head and upper extremities in a relaxed position and looking straight ahead^10^[12]. The Gyko^®^ system (Microgate, Italy)^11^ was used to record the elliptical area of oscillation (EA in cm2) for 60 s in the standing position. The Bluetooth-enabled sensor transmits real-time data to specialist software, which automatically calculates EA as a measure of balance. This protocol provides moderate-to-strong evidence of validity and reliability^12,13^. The sensor was placed on the participant’s mid-back using a harness. The evaluation was performed in the following order^13,14^: eyes open, eyes closed on a rubber surface, and eyes closed on a stable surface. The participants kept their gaze fixed at a reference point that was 2 m away. All measurements were performed under standardized conditions. The participants remained barefoot, wearing comfortable clothing and without accessories. A specialized model of intermediate-density rubber (Balance Pad Elite^®^) was used for the balance test on an unstable surface. This rectangular device (50 cm × 41 cm × 6 cm) weighing 0.7 kg has a nominal density of 55 kg/m3 and vertical resistance of 0.45 N/mm2. These properties make it suitable for moderately increasing the instability during standing.

#### Physical activity questionnaire for children (PAQ-C)

The Spanish version of the validated Physical Activity Questionnaire for Children (PAQ-C)^15^ will be used to collect data on the type and amount of physical activity performed by the participants.

#### Lunge test

**Fig. 2:**
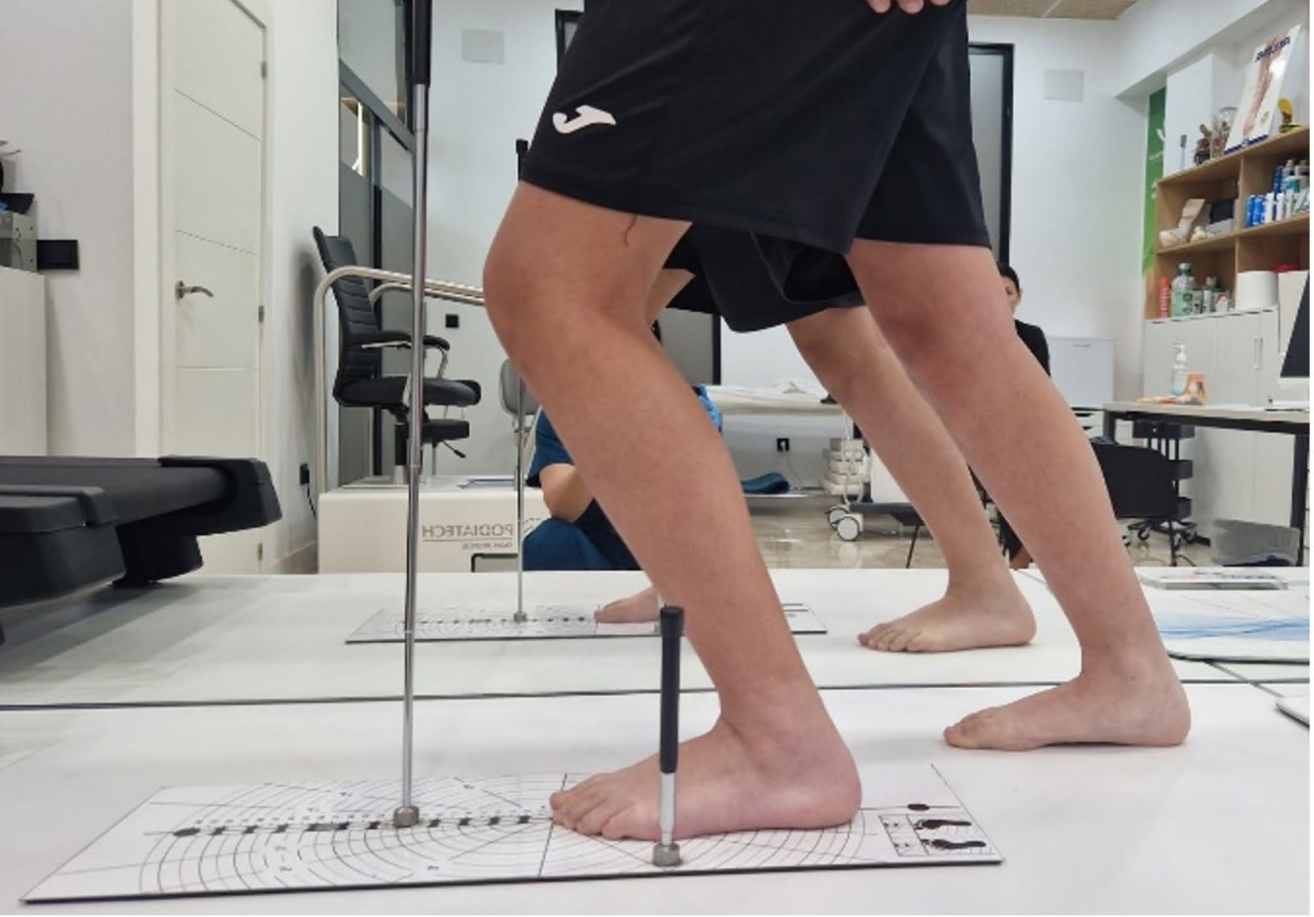
The figure represents the final position during the execution of the Lunge test.

Dorsal flexion of the ankle was evaluated using the lunge test (Fig. 2). If the distance from the wall to the third metatarsal was less than 10 cm, this would be considered a normal result^11^. Initially, ankle range of motion was assessed using the Leg Motion system (Check Your Motion ^®^, Albacete, Spain). This test evaluates active ankle dorsiflexion in the standing position. The final score (cm) of the test was the distance between the first metatarsal and the wall when the participant flexed the knee towards the vertical position without lifting the heel^16^. To familiarize themselves with the test, participants performed 3–4 attempts following the therapist’s instructions: (a) do not lift the heel off the surface; (b) try to bring your knee as far as possible towards the midline; and (c) do not compensate the movement using your arms. The highest value of three to 3–4 attempts was used in the analysis^17^.

### Interventions

**Fig. 3:**
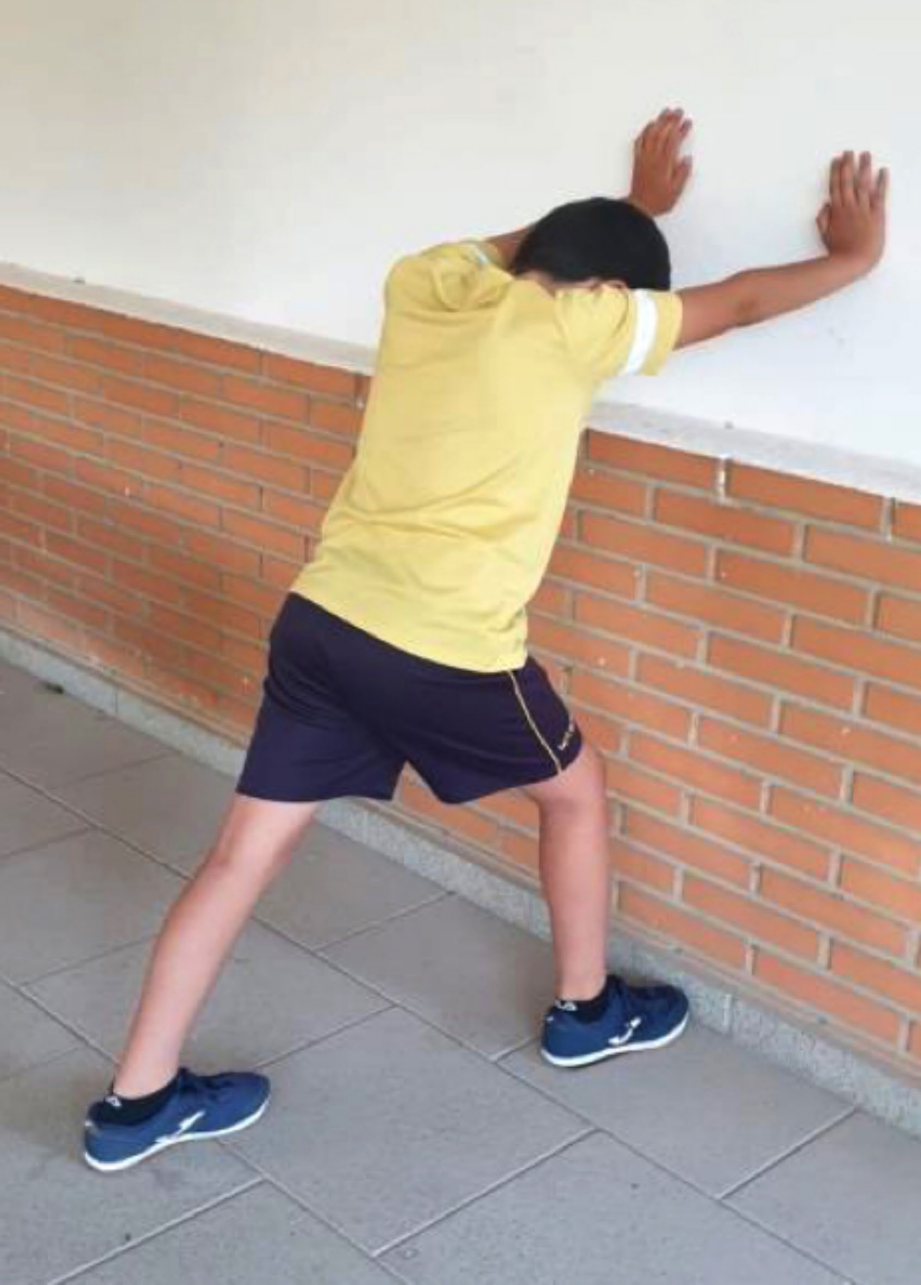
The figure represents the position held during the global stretch of the triceps surae.

In the stretching group, participants embarked on a stretching exercise routine. The stretching exercise involved taking a big step forward with one leg while keeping the other leg extended backward with the knee bent, with the aim of improving flexibility and range of motion. When adopting this position, one aims to keep the trunk upright and the front foot fully flat on the ground, with the knee bent at a 90-degree angle and aligned with the ankle. Meanwhile, the back leg remained extended, with the knee and hip at 90-degree angles not touching the ground (Fig. 3). The hip is lowered toward the floor, keeping the back straight and preventing the front knee from going over the toes, which causes intense elongation in the front of the thigh and back of the rear leg. The position was held for 15–30 s while breathing deeply to allow the muscles to relax and elongate. The exercise was repeated with the other leg forward, alternating between the two to progressively improve flexibility and range of motion of the leg and hip muscles. These exercises were performed three times a day, with ten repetitions per exercise, and were recorded on a control sheet.

In the heel cup group, a specific intervention was applied in the form of 5 mm heel cups. These heel cups, made of 65 Shore A hardness EVA (Ethyl Vinyl Acetate), are designed to provide additional support to the heel, which in turn helps to keep the musculature relaxed by bringing the origin and insertion closer together. By reducing stress and pressure on the heel, the heel cups are expected to facilitate a more balanced posture and a smoother, pain-free gait. This group will wear the heel cups continuously throughout the study period, allowing for a full evaluation of their potential impact on foot function.

### Sample size calculation

The averages of two normal populations were compared, assuming that the standard deviations of both populations are equal, with a value of 0.9 units A priori sample size calculation was performed using the difference between means of two independent groups using G*Power 3.1.9.2 software, two-tailed hypothesis, a mean effect size of 0.50, a probability of error α of 0.05, with a β level of 15% and a desired power analysis of 85% (probability of error 1-β) were used for sample size calculations. Therefore, a total sample size of 59 participants per group was calculated. Assuming a loss of 20% it was assumed that a minimum of 73 observations were required in each sample. In total, there were 146 observations. The expected half-width of the Confidence Interval is 0.206 units.

### Statistical analysis

Data were summarized using the mean (standard deviation) and median (1st and 3rd quartiles) for numerical variables and using absolute frequency (relative frequency) for qualitative variables. General descriptive data were also presented, over time, and over time during treatment, 95% confidential interval. A multivariable approach was used for the analysis. Univariable analyses were discarded to avoid biases and to prevent amplification of Type I errors (false positives). To compare the evolution of stabilometry over time, and its differences between the two treatment groups, a linear mixed regression model was fitted for each test. To minimize the influence of scales with skewed distributions, the stabilometry scales were log-transformed. The double interaction between time and treatment group was considered. The analyses were performed using the statistical program R (v 4.1.2) and the libraries lme4 (v 1.1–27.1), ggplot2 (v 3.3.5), clickR (v 0.8.0), repmod (v 0.1.7), DHARMa (v 0.4.4), and ggeffects (1.1.1).

## Results

The stabilometry values for the three periods are listed in Table 2. The data were described using the median. Table 3 shows the descriptive data for time and treatment. This shows the evolution of the stabilometry over time for each treatment.

**Table 2:** Descriptive data over time.

Variable	Basal *N* = 150	3 months *N* = 150	6 months *N* = 150
	Mean (SD) / n(%)	Mean (SD) / n(%)	Mean (SD) / n(%)
	Median (1st, 3rd Q.)	Median (1st, 3rd Q.)	Median (1st, 3rd Q.)
EAcm2OA	54.05 (31.23)	53.5 (29.6)	51.48 (28.11)
	47.45 (30.04, 68.92)	48.18 (30.48, 68.27)	46.63 (28.99, 65.61)
EAcm2OAtto	44.85 (23.81)	44.52 (23.62)	45.34 (23.3)
	39.99 (25.45, 57.27)	39.5 (25.45, 55.34)	40.57 (26.02, 58.33)
EAcm2OC	66.1 (33.87)	66.25 (34.12)	64.8 (32.72)
	57.78 (40.77, 85.78)	59.98 (38.83, 85.35)	59.94 (39.61, 82.55)
EAcm2OCtto	56 (25.28)	55.53 (25.2)	56.68 (26.2)
	52.49 (34.58, 71.65)	51.42 (34.24, 68.98)	54.43 (34.76, 72.5)
EAcm2EOA	78.7 (37.02)	77.83 (35.88)	76 (33.75)
	70.25 (49.94, 97.23)	70.81 (48.8, 96.1)	70.15 (49.37, 96.4)
EAcm2EOC	91.82 (40.71)	89.88 (39.61)	88.59 (36.87)
	82.46 (63.5, 107)	81.92 (62.42, 103.23)	80.6 (61.29, 106.12)

Both groups EAcm2OA (area of ellipse in square centimeter with Eyes Open), EAcm_2_OC (area of ellipse in square centimeters with Eyes Closed), EAcm_2_EOA (area of ellipse in square centimeters with Eyes Open on foam surface), EAcm_2_EOC (area of ellipse in square centimeters with Eyes Closed on foam surface.

**Table 3:** Descriptive data over time and during treatment.

Variable	Exercises baseline *n* = 76 Mean (SD)/*n*(%)	Basal heel cup *n* = 74 Mean (SD)/*n*(%)	Exercises 3 months *n* = 76 Mean (SD)/*n*(%)	Heel cup 3 months *n* = 74 Mean (SD)/*n*(%)	Exercises 6 months *n* = 76 Mean (SD)/*n*(%)	Heel cup 6 months *n* = 74 Mean (SD)/*n*(%)
EAcm2OA (cm^2^)	55.9 (32.97)	52.16 (29.45)	53.31 (29.86)	53.69 (29.54)	45.22 (26.42)	57.9 (28.52)
	50.06 (33.82, 62.88)	46.86 (27.31, 70.95)	48.39 (32.17, 62.2)	47.52 (29.22, 72.41)	40.58 (26.23, 52.14)	50.86 (37.7, 77.06)
EAcm2OC (cm^2^)	67.15 (32.68)	65.02 (35.25)	65.24 (31.09)	67.29 (37.16)	55.84 (26.95)	73.99 (35.63)
	59.88 (42.49, 86.87)	57.57 (34.97, 85.73)	58.12 (39.45, 83.28)	59.98 (37.92, 86.78)	50.24 (34.69, 65.86)	68.12 (46.71, 93.66)
EAcmEOA (cm^2^)	79.7 (37.11)	77.66 (37.16)	76.86 (34.18)	78.83 (37.76)	68.53 (29.51)	83.67 (36.23)
	70.81 (51.86, 96.71)	68.77 (49.02, 97.44)	71.06 (48.86, 94.13)	70.81 (49.09, 97.44)	61.67 (44.25, 86.74)	79.65 (55.82, 100.78)
EAcmEOC (cm^2^)	92.98 (39.84)	90.63 (41.82)	89.89 (37.14)	89.87 (42.26)	80.74 (30.75)	96.65 (40.91)
	82.37 (64.98, 103.9)	82.46 (59.78, 108.18)	80.41 (63.8, 101.45)	84.73 (60.26, 106.57)	72.04 (60.32, 92.35)	93.48 (65.37, 113.78)
FXD.Derch (grade)	5.88 (2.59)	6.28 (2.09)	6.28 (2.41)	6.28 (2.09)	7.7 (1.89)	6.28 (2.09)
	5.5 (3, 8)	7 (5, 8)	6 (4, 8.25)	7 (5, 8)	8 (6.75, 9)	7 (5, 8)
FXD.Left (grade)	5.83 (2.62)	6.05 (2.18)	6.26 (2.41)	6.05 (2.18)	7.67 (1.91)	6.05 (2.18)
	5.5 (3, 8)	7 (5, 8)	6 (4, 8.25)	7 (5, 8)	8 (6, 9)	7 (5, 8)

Data represent mean ± sd. Differences among groups were analyzed by ANOVA.

The linear mixed regression model applied to the variable Ellipse Area Extent in Square centimeters with Eyes Open (EAcm2OA) provided strong evidence that heel cup use was significantly correlated with an increase (i.e., worsening) in this measure of postural control and balance. Specifically, it was estimated that heel cup use was correlated with a 4% increase in EAcm2OA at 3 months compared with no heel cup use (95% CI: 0.9–7%, *p* = 0.011). The negative effect of heel cups was even greater at six months, with an estimated 16% increase in EAcm2OA (95% CI: 11.9–18%, *p* < 0.001). In contrast, the stretching exercise program showed a significant correlation with reduction (i.e., improvement) in EAcm2OA. With exercise, a reduction in EAcm2OA of 1.7% at 3 months (95% CI: -3.4% to -0.13%, *p* = 0.011) and a further reduction of 12.5% at 6 months (95% CI: -12.7% to -8.7%, *p* < 0.001) was estimated.

When evaluating the more demanding eye closed condition (EAcm2OC), similar patterns were observed. Heel cup use was again associated with a significant increase in ellipse area, which was 3.4% at 3 months (*p* = 0.026) and 18% at 6 months (*p* < 0.001), indicating a detrimental effect on postural control measured with eyes closed. In contrast, stretching exercises showed a non-significant reduction of 1% at 3 months and a significant reduction of 5.5% at 6 months (*p* < 0.001) in EAcm2OC, indicating improvements in balance with the eyes closed.

Even in the more difficult condition of eyes closed on an unstable foam surface (EAcm2EOC), the pattern of negative results from heel cups and positive results from exercise was maintained. Specifically, in the EAcm2EOC group, exercise was associated with a significant reduction of 5% at 6 months (95% CI: -6.4%–-1.9%, *p* < 0.001). In contrast, the use of heel cups showed a significant increase of 8% in this measure at 6 months (95% CI: 3.9–12%, *p* < 0.001).

These findings under different conditions (eyes open/closed and stable/unstable surface) provide consistent and convergent evidence. The use of heel cups seems to significantly impair postural control in children with triceps surae retraction, whereas the incorporation of a simple stretching exercise program also generates significant improvements in these fundamental variables for adequate motor performance.

Beyond the differential effects of these two treatments, the statistical analysis also sheds light on the role of regular physical activity in the postural control of these children. It was consistently observed in all models that higher levels of physical activity were closely associated with reductions in ellipse areas, indicating a beneficial effect on balance. For example, in the eyes-open condition, children with moderate physical activity levels experienced a 28.8% reduction in EAcm2OA compared with children with very low activity levels (95% CI: -43.8% − 13.6%, *p* < 0.001). Greater reductions were observed in children with high and very high physical activity levels (reductions of 49.9% and 74.5%, respectively, *p* < 0.001).

Sex was another demographic factor that was closely correlated with stabilometric measurements. Across the various models, it was consistently evident that male children showed significant increases in ellipse areas compared with female children. For example, with eyes open, boys showed a 44.1% increase in EAcm2OA compared to girls (95% CI: 33.3–54.9%, *p* < 0.001). This indicates a beneficial effect of being female on postural control in this sample. The age of the participants, between 8 and 12 years, did not exhibit any significant correlation with the stabilometric measurements. There were also no close associations between these measurements and the degree of ankle dorsiflexion (Table 3).

**Fig. 4:**
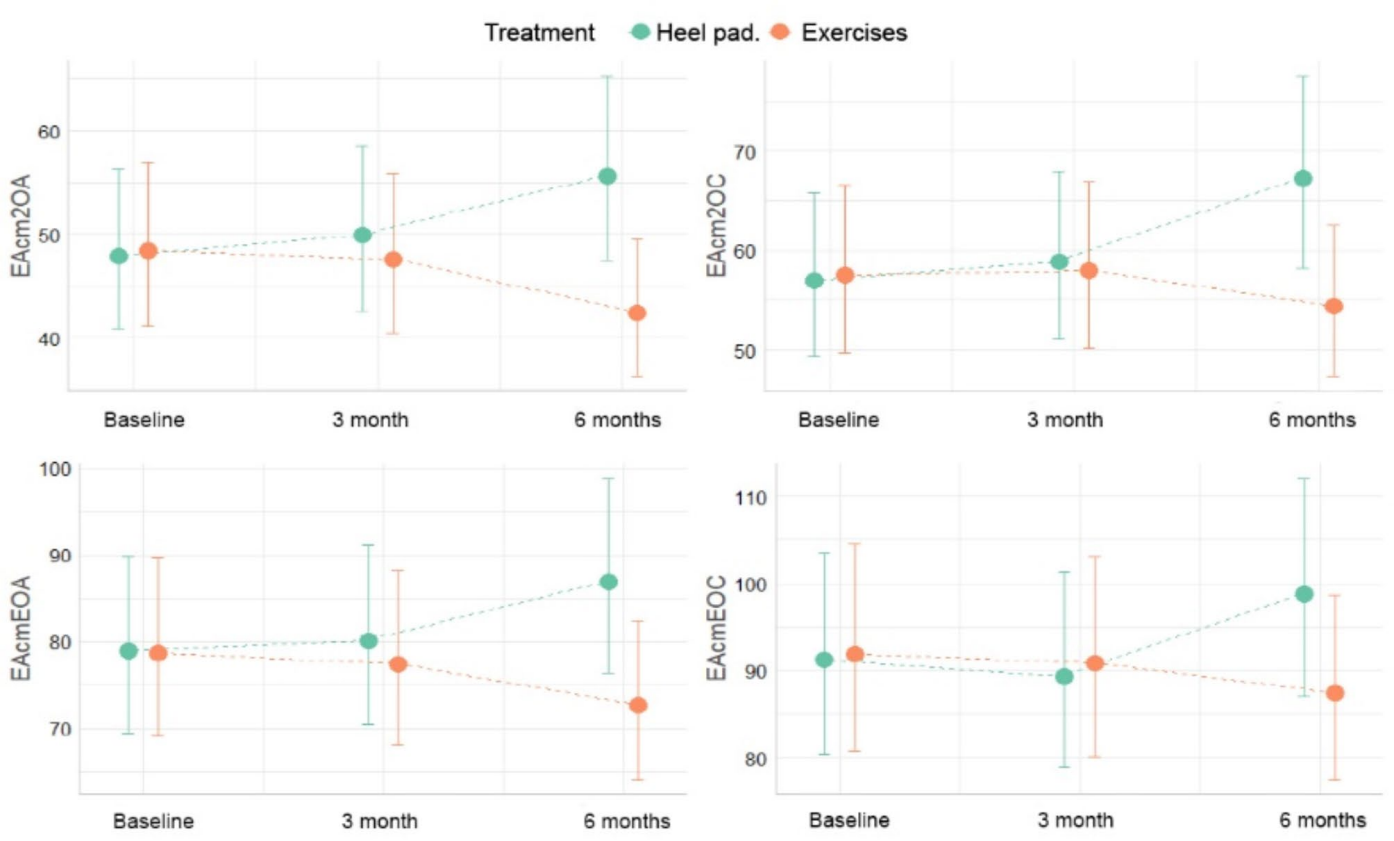
The figure represents the effect of each intervention on the data points collected.

Figure 4 illustrates the conditional effects. In this figure, we can see the average estimated evolution along with the confidence intervals for each treatment. It can be seen that, from the baseline measurement at 3 months, there is no notable evolution in any group; however, at 6 months, the differences between heel cup and exercise are notable. Exercises improve stabilometry (reduce the area), while heel cups tend to worsen it. The results of the hypothesis testing of both treatments at 3 and 6 months showed significant differences in the comparison of the means of the stabilometric variables between both groups of different treatments. Table 4 shows an example of the EAcm2OA condition with heel pad; the ellipse area increased by 4% at 3 months and 16% at 6 months, while with exercises it decreased by 30% at 6 months.

**Table 4:** Predictions with 95%CI for stabilometric variables.

Variable	Time of treatment	Heel cup		Exercises	
		Prediction	95% CI	Prediction	95% CI
EAcm2OA	Basal	47.91	[40.84; 56.20]	48.35	[41.09; 56.90]
	3 months	49.86	[42.50; 58.49]	47.52	[40.46; 55.81]
	6 months	55.64	[47.43; 65.27]	42.34	[36.21; 49.50]
EAcm2OC	Basal	56.97	[49.37; 65.74]	57.55	[49.73; 66.60]
	3 months	58.90	[51.04; 64.97]	57.97	[50.19; 66.95]
	6 months	67.19	[58.23; 77.54]	54.38	[47.29; 62.54]
EAcm2EOA	Basal	78.99	[69.47; 89.82]	78.70	[69.03, 89.71]
	3 months	80.09	[70.43, 91.07]	77.44	[68.05, 88.14]
	6 months	86.88	[76.40, 98.79]	72.70	[64.11, 82.45]
EAcm2EOC	Basal	91.20	[80.39; 103.47]	91.89	[80.80, 104.51]
	3 months	89.39	[78.80, 101.41]	90.89	[80.11, 103.12]
	6 months	98.78	[87.08, 112.07]	87.46	[77.50, 98.71]

Adjusted for a 10-year-old woman with very low PAQC and ankle flexion 7. Example for the EAcm2OA condition for the three and six months.

## Discussion

The results of this study indicate that stretching exercises for children aged 8 to 12 with retraction of the posterior muscles (gastrocnemius and soleus) significantly improve postural stability and control, as measured after six months of treatment. Clinically, these findings suggest that routine use of heel cups in children with triceps surae retraction may worsen postural control and balance. Conversely, a simple stretching program yields substantial improvements in these essential motor functions. Additionally, children who engage in more physical activity exhibit better stabilometric outcomes, with girls showing superior postural control compared to boys. This underscores the importance of an active lifestyle for enhancing postural control and balance in children.

Limited research exists on postural control and gastrosoleus retraction. However, restricted ankle dorsiflexion impairs functional activities and may lead to recurrent ankle sprains^18,19^. Our study found no significant differences in children with limited dorsiflexion, though increased ellipse area values indicated reduced stabilometric control, correlating with poorer postural control in static and dynamic states^20^. This suggests a link between postural control issues and sports-related injuries, particularly chronic ankle instability. The Lunge Test assessed dorsal ankle flexion, showing that improving dorsiflexion in patients with chronic instability reduces injury risk and enhances postural control^21–23^. Comprehensive rehabilitation, including balance training and stretching the talocrural joint, promotes postural improvements^24^. Our findings align with current studies, demonstrating that enhancing ankle flexion through posterior muscle stretching increases stability under various conditions (OA, OC, EA, and EC).

Our study showed similar results in children aged between 8 and 12 years with stretching; enhanced muscle stretching improved postural control. Mohamed (2015)^25^ shows that vibration training improved postural control and muscle strength in children after a 6-month training program. In the present study, after six months of daily stretching. In addition, other important data found in our research show that the use of a 5 mm heel cup does not improve postural control; therefore, this should be considered in orthopedic treatments that are incorporated in plantar orthoses or placed individually. Few studies have investigated postural control and muscle activity in orthopedic footwear^26^. This demonstrates the importance of assessing the gastrocnemius-soleus musculature, stiffness, and length^26,27^ when it comes to postural control. These contributions should be considered in future orthopedic treatments and open the door to future lines of research.

### Study limitations

This study had some limitations that should be considered. Adherence to exercise programs as well as the use of heel cups was based on reports from participants and their parents, which could introduce bias. Moreover, the subjective assessment of symptoms or health-related quality of life was not included. Biomechanical or neurophysiological measurements that could fully clarify the mechanisms of action have not been performed. Additionally, follow-up was limited to 6 months; therefore, studies of longer duration would be required to evaluate long-term effects. Another limitation is that the maturity status (puberty stages) of the participants was not assessed. Physiological maturation can influence postural control and response to training, and the absence of this measurement may have introduced variability in the results. Future studies should consider including an evaluation of maturity status to better understand its potential impact on the effects of interventions aimed at improving postural control in children aged 8 to 12 years.

## Conclusions

Exercise performance in children with retraction of the posterior musculature (triceps surae), performed on a constant basis, improves postural control in children with gastrosole retraction. Heel cup treatments should be scrutinized in orthopedic treatments, as they may cause deterioration in postural control over time in patients with retraction. Despite these limitations, this study provides preliminary evidence regarding the management of pediatric patients.

## Electronic supplementary material

Below is the link to the electronic supplementary material.

Supplementary Material 1

## Data Availability

The datasets used and/or analysed during the current study are available from the corresponding author on reasonable request.
