# Supplementary material for: Postural analysis of children with muscle retraction after six-month exercise or heel cup interventions in a randomized trial
Source: Sci Rep. 2025 Apr 19;15:13578. doi: 10.1038/s41598-025-98527-6 (PMC12009426; doi:10.1038/s41598-025-98527-6)
Supplement: Supplementary file 1 — Supplementary Material 1 [file 41598_2025_98527_MOESM1_ESM.pdf]

Supplementary material.

Table 3 Summary of the linear mixed regression model for AEcm2OA. The table presents the fixed factors of the model together with the time x TTO interaction. The estimates are in logarithmic scale, so they cannot be interpreted directly.

Variables	***Estimate***	***Std Error***	***Lower 95%***	***Upper 95%***	***P.value***
(Intercept)	3.939	0.222	3.514	4.366	<0.001
3 months: heel cup	0.040	0.016	0.009	0.07	0.011
6 months: heel cup	0.150	0.023	0.119	0.180	<0.001
Exercises vs. heel cup (basal)	0.009	0.05	-0.087	0.105	0.855
Low vs. very low activity	-0.192	0.081	-0.347	-0.035	0.02
Moderate Activity vs. very low	-0.288	0.079	-0.438	-0.136	<0.001
High vs. very low activity	-0.499	0.083	-0.658	-0.338	<0.001
Very high vs. very low activity	-0.745	0.101	-0.937	-0.551	<0.001
Man	0.441	0.056	0.333	0.549	<0.001
Age	0.025	0.02	-0.014	0.064	0.218
FXD.Right	-0.005	0.028	-0.06	0.051	0.872
FXD.Left	-0.04	0.028	-0.095	0.014	0.159
3 months: exercises	-0.057	0.022	-0.1	-0.013	0.011
6 months: exercises	-0.282	0.028	-0.335	-0.227	<0.001
Sd id(Intercept)	0.287				
Residual	0.095				

Table 4 Summary of the linear mixed regression model for EAcm2OC. The table presents the fixed factors of the model together with the time x TTO interaction. The estimates are in logarithmic scale, so they cannot be interpreted directly.

Variables	***Estimate***	***Std Error***	***Lower 95%***	***Upper 95%***	***P.value***
(Intercept)	4.603	0.199	4.222	4.985	<0.001
3 months: heel cup	0.033	0.015	0.004	0.062	0.026
6 months: heel cup	0.165	0.021	0.136	0.194	<0.001

Exercises vs. heel cup (basal)	0.01	0.045	-0.076	0.096	0.823
Low vs. very low activity	-0.045	0.073	-0.184	0.095	0.542
Moderate activity vs. very low	-0.179	0.07	-0.314	-0.044	0.012
High vs. very low activity	-0.503	0.075	-0.646	-0.36	<0.001
Very high vs. very low activity	-0.509	0.09	-0.682	-0.336	<0.001
Man	0.358	0.05	0.262	0.455	<0.001
Age	-0.01	0.018	-0.045	0.024	0.564
FXD.Right	-0.006	0.027	-0.058	0.046	0.818
FXD.Left	-0.058	0.026	-0.109	-0.007	0.029
3 months: exercises	-0.026	0.021	-0.067	0.015	0.22
6 months: exercises	-0.222	0.026	-0.272	-0.171	<0.001
Sd id(Intercept)	0.255				
Residual	0.09				

Table 5 Summary of the linear mixed regression model for EAcm2EOA. The table presents the fixed factors of the model together with the time x TTO interaction. The estimates are in logarithmic scale, so they cannot be interpreted directly.

Variables	***Estimate***	***Std Error***	***Lower 95%***	***Upper 95%***	***P.value***
(Intercept)	4.77	0.179	4.428	5.113	<0.001
3 months: heel cup	0.014	0.013	-0.011	0.038	0.270
6 months: heel cup	0.095	0.018	0.071	0.012	<0.001
Exercises vs. heel cup (basal)	-0.004	0.04	-0.081	0.073	0.927
Low vs. very low activity	-0.131	0.065	-0.256	-0.005	0.048
Moderate activity vs. very low	-0.304	0.063	-0.425	-0.182	<0.001
High vs. very low activity	-0.534	0.067	-0.662	-0.405	<0.001
Very high vs. very low activity	-0.557	0.081	-0.712	-0.401	<0.001
Man	0.317	0.045	0.23	0.404	<0.001
Age	-0.017	0.016	-0.049	0.014	0.286
FXD.Right	-0.014	0.023	-0.058	0.031	0.552
FXD.Left	-0.019	0.023	-0.064	0.024	0.392
3 months: exercises	-0.03	0.018	-0.064	0.024	0.095
6 months: exercises	-0.174	0.022	-0.217	-0.13	<0.001
Sd id(Intercept)	0.231				
Residual	0.076				

Table 6 Summary of the linear mixed regression model for EAcm2EOC. The table presents the fixed factors of the model along with the time x TTO interaction. The estimates are in logarithmic scale so, they cannot be interpreted directly.

Variables	***Estimate***	***Std Error***	***Lower 95%***	***Upper 95%***	***P.value***
(Intercept)	5.078	0.173	4.748	5.409	<0.001
3 months: heel cup	-0.02	0.021	-0.06	0.02	0.33

					4
6 months: heel cup	0.08	0.026	0.039	0.12	<0.001
Exercises vs. heel cup (basal)	0.008	0.041	-0.07	0.085	0.853
Low vs. very low activity	-0.105	0.063	-0.225	0.015	0.096
Mod activity vs. very low	-0.25	0.061	-0.367	-0.133	<0.001
High vs. very low activity	-0.478	0.065	-0.602	-0.353	<0.001
Very high vs. very low activity	-0.494	0.079	-0.644	-0.342	<0.001
Man	0.257	0.043	0.175	0.339	<0.001
Age	-0.027	0.016	-0.057	0.003	0.087
FXD.Derch	-0.002	0.031	-0.061	0.057	0.942
FXD.Left	-0.039	0.03	-0.098	0.018	0.191
3 months: exercises	0.009	0.029	-0.048	0.066	0.757
6 months: exercises	-0.129	0.033	-0.193	-0.064	<0.001
Sd id(Intercept)	0.21				
Residual	0.126				
